# Supplementary material for: Neurofilaments as Emerging Biomarkers of Neuroaxonal Damage to Differentiate Behavioral Frontotemporal Dementia from Primary Psychiatric Disorders: A Systematic Review
Source: Diagnostics (Basel). 2021 Apr 22;11(5):754. doi: 10.3390/diagnostics11050754 (PMC8146697; doi:10.3390/diagnostics11050754)
Supplement: Supplementary file 1 [file diagnostics-11-00754-s001.zip › diagnostics-1161805-supplementary.pdf]

Table S1. Aggregated summary of literature review.

	References	Number of Participants				Neurofilament (pg/mL)						Age						Diagnostic Subgroups									
						FTD		PPD		Controls		FTD		PPD		Contrôles		FTD					PPD				
		Total	bvFTD	PPD	Control	Mean	SD	Mean	SD	Mean	SD	Mean	SD	Mean	SD	Mean	SD	Poss.	Prob.	Def.	bvFTD	Other	Depr	BP	Schiz	Anxie	Others
CSF	Vivjverberg et al. (2017)	47	22	25		9660	6739	857	299			63	6	61	7			0	19	3	0		11	4	0	1	9
	Rolstad et al. (2015)	153		82	71			486	426	254	55			38	15	38	13						0	82	0	0	0
	Isgren et al. (2017)	77		77				395	826					40	17								0	77	0	0	0
	Jakobsson et al. (2015)	219		133	86			480	288	359	315			36	13	38	17						0	133	0	0	0
	Fourier et al. (2020)	114	50	64		4442	3427	1199	745			60	11	64	15			0	0	17	33		36	8	3	12	5
	Eratne et al. (2019)	52		31	21			949	379	1036	282			66	3	51	9						9	9	9	1	3
	Scherling et al. (2014)	92	45		47	5225	4753			1164	627	65	8			66	11	0	0	0	45						
	Magdalinou et al. (2015)	46	16		30	1428	981			619	319	63	9			60	6	0	0	0	16						
	Gaiani et al. (2017)	64	20		44	2337	1321			507	371	65	8			54	24	0	0	0	20						
	Alcolea et al. (2017)	144	68		76	2174	2395			461	220	67	9			60	8	0	0	0	68						
	Lista et al. (2017)	30	9		21	1050	649			633	204	73	3			64	7	0	0	0	9						
	Abu-Rumeileh et al. (2018a)	90	52		38	4587	5344			1283	649	65	10			63	8	0	0	0	52						
	Ljubenkov et al. (2018)	89	40		49	5610	4800			1300	610	63	8			64	8	0	0	0	40						
	Goosens et al. (2018)	66	46		20	4557	3514			1889	2742	64	12			69	10	0	0	0	30	6					
	Wilke et al. (2016)	87	41		46	2495	1081			1044	457	68	11			66	11	0	0	0	41						
	Abu-Rumeileh et al. (2018b)	57	19		38	5846	8002			1172	569	63	9			64	11	0	0	0	19						

	Meeter at al. (2018)	224	179		45	3246	2291			982	567	63	8			60	9	0	0	0	179						
	Paterson et al. (2018)	75	45		30	1988	1910			671	260	62	7			64	13	0	0	4	41						
	Hampel et al. (2018)	30	9		21	1050	649			633	204	72	3			64	8	0	0	0	9						
	Niikado et al. (2019)	24	13		11	2610	1685			657	287	59	6			64	6	0	0	0	13						
	Steinacker et al. (2018)	37	37			2948	2630					64	9					15	17	5	0						
	Abu-Rumeileh et al. (2020)	58	30		28	3111	3560			891	264	67	8			65	10	0	0	22	0	7					
	Antonell et al. (2020)	100	50		50	2617	1854			406	137	62	9			57	13	0	0	0	40	8					
**SERUM**	Besse et al. (2019)	30		15	15			17	12	16	0			49	14	49	14						0	15	0	0	0
	Rami Al Shweiki et al. (2019)	97	20	50	27	64	49	17	7	15	6	49	6	52	9	47	12	9	5	6	0		28	11	11	0	0
	Katisko et al. (2020)	100	66	34		37	33	16	10			64	9	56	9			0	66	0	0		19	8	7	0	0
	Wilke et al. (2016)	87	41		46	50	38			19	18	68	11			64	11	0	0	0	41						
	Rohrer et al. (2016)	62	34		28	58	33			20	8	63	8			64	7	0	0	0	34						
	Verde et al. (2019)	62	12		50	56	70			34	57	64	23			60	34	0	0	2	10						
	Steinacker et al. (2018)	89	74		15	49	35			22	21	64	9			65	11	15	17	5	0						
	van der Ende et al. (2019)	186	59		127	48	34			8	4	63	8			49	15	0	0	59	0	17					
	Vivjverberg et al. (2017)	47	22	25		9660	6739	857	299			63	6	61	7			0	19	3	0		11	4	0	1	9
